# The Association Between Facial Dermatosis and Face-Mask Wearing During COVID-19 in Saudi Arabia

**DOI:** 10.7759/cureus.22265

**Published:** 2022-02-15

**Authors:** Hatoon M Althobaiti, Hend Althobaiti, Muhammad Khan, Hanadi Alsatti, Sahal J Samarkandy

**Affiliations:** 1 College of Medicine, King Saud Bin Abdulaziz University for Health Sciences, Jeddah, SAU; 2 Medical Education, King Saud Bin Abdulaziz University for Health Sciences, Jeddah, SAU; 3 Dermatology, King Abdulaziz Medical City (KAMC) Western Region, Jeddah, SAU

**Keywords:** mask wearing, pandemic, facial dermatosis, face mask, covid-19

## Abstract

Background: During the coronavirus pandemic of 2019, there has been an upsurge in the number of reported cases of facial dermatosis caused by face masks wearing within the general population. Face mask-induced facial dermatosis has been investigated previously in healthcare workers without involving the general population. However, as a precautionary measure against the coronavirus disease 2019 (COVID-19) pandemic, wearing a face mask has become mandatory for the general population, similar to healthcare workers.

Objective: To measure the prevalence of COVID-19 pandemic-induced facial dermatosis. Also, to determine the type of face mask used that causes the most facial dermatosis and the association between prolonged usage of face masks and facial dermatosis in Western Saudi Arabia's population.

Methods: The study covers the Western region of Saudi Arabia. A self-administered Google survey was shared on social media. The study used prior surveys from similar studies. The data collection included participants’ demographic information, pre-existing skin conditions, mask type, and mask-related skin conditions. The data were analyzed using the statistical package for the social sciences (SPSS) version 20.0 (IBM Corp., Armonk, NY).

Results: The median age was 30 years (interquartile range {IQR} 23-43). Females represented 65.5% of our sample. Face mask-wearing was associated with skin changes in 41.7% of the population. The most common skin condition associated with mask use was pimples and pustules representing 28.7%, of which the most common site was on the cheeks 31.4%. Acne vulgaris was the most common pre-existing skin condition, affecting 8.7% of the total population. Of the total, 46.2% experienced an exacerbation of their pre-existing skin condition with or after wearing masks. Skin changes were significantly associated with skin type and duration of wearing the mask (P<0.001).

Conclusion: The prevalence of facial mask-induced facial dermatosis is 41.7% of the general population in which surgical mask was responsible for most cases. In addition, there is a strong association between the duration of wearing the mask and facial dermatosis.

## Introduction

In March 2020, the World Health Organization (WHO) announced that coronavirus disease 2019 (COVID-19) has become a global pandemic after spreading to 114 countries of the world at that time [1]. By then, the whole world was forced to implement the necessary precautions to prevent the further spread of the virus by washing hands and wearing personal protective equipment (PPE) specially face mask according to the guidelines of the Center for Disease Control and Prevention (CDC) [2]. Face masks were first presented to the world in 1897 for surgical procedures use [3]. Then in 2003, the use of face masks by the general population started during the severe acute respiratory syndrome (SARS) pandemic, and since then their great value was seen in providing protection from human-to-human respiratory viral transmissions [4]. Secondary to the obligated and prolonged use of face mask during the COVID-19 pandemic, there has been an increase in the reported cases of facial dermatosis such as rosacea, acne, and seborrhoeic dermatitis [5-7]. Common inflammatory facial dermatosis is defined as skin lesions affecting the face, it is a group of dermatological conditions of the skin including “acne vulgaris (AV), papulopustular rosacea (PPR), erythematotelangiectatic rosacea (ETR), perioral dermatitis, seborrheic dermatitis (SD), and atopic dermatitis (AD) [8]. It has been suggested in previous studies that prolonged wearing of face mask increases friction, occlusion, and causes hyperhydration which leads to a breach in the epidermal integrity of the skin, thus causing the apparent damage which manifests as facial dermatosis [9].

A recent study performed in China documented a rate of 97% of skin damage was caused by using personal protective equipment (PPE) such as N95 masks among the first line healthcare workers [10]. Also, a similar study reported different types of facial dermatoses caused by PPE among healthcare workers during the COVID-19 pandemic, where they found that irritant contact dermatitis was the most common reported facial dermatosis [9]. In India, a recent study showed a 56% increase of face acne after the prolonged wear of face masks among healthcare workers [11]. All these studies were focusing on the healthcare providers without including the general population. However, due to COVID-19 pandemic obligations, the general population had engaged in the face mask wearing similar to health care providers. 

Up to our knowledge, there are limited studies investigating the association between facial dermatosis and face mask wearing among the general population during COVID-19 pandemic. An exception to this is a study that was conducted in Poland which evaluated the prevalence and factors contributing to face mask induced itch only, without exploring other facial dermatoses [12].

Currently, there is a lack of studies that investigate facial mask induced facial dermatosis among the general population. Moreover, high temperature and humidity act as a precipitating factor with flaring of acne and sebum excretion [6]. We assume a higher incidence in Saudi Arabia, specifically the Western region since it is known to have hot and humid climate. Hence, we focus on the importance of conducting this study among the general population in Western region of Saudi Arabia to find the association between the prolonged wearing of face mask and facial dermatoses during COVID-19 pandemic.

## Materials and methods

Design/setting

Our study is an observational cross-sectional study that was conducted through a self-administered Google survey delivered electronically via social media link.

Area and population

The study was conducted among the general population in the Western region of Saudi Arabia. The website www.raosoft.com/samplesize.html provided Raosoft® software was used to compute the sample size. Saudi Arabia has a population of approximately 34,2018,169 people in 2019. The required sample size was estimated at the 95% confidence level with an estimated 60.4% prevalence of itch by using facial masks with a margin of error of 5%. The required minimum sample size was determined to be 368. Our study targeted a larger sample size to ensure that it is representative of the Western region. Our sample consisted of 446 participants who were above 18 years and use any of the following: surgical mask, cloth mask, cone-style mask, KN95 masks, respirator with filtering piece, or face shield with surgical mask during 2021. We excluded those who do not wear face masks, wear double masks, face shield without wearing face masks, or Niqab, a veil that Muslim women use to cover their face.

Data collection and statistical analysis: Data was collected using an online self-administered Google survey. The questionnaire was created using surveys from previous similar studies that contained questions related to demographic data, baseline skin conditions, type of masks, and mask-related skin conditions [12,13]. The survey was translated to Arabic, and pictures of different face mask types were attached as an option to be selected in the study. Data was through Google survey on Microsoft Excel, and after all the data was collected, it was transferred to SPSS statistics, version 20.0 (IBM Corp., Armonk, NY) for analysis. Quantitative characteristics, including age, weight, and height, were analyzed by estimating the standard deviation and mean. We calculated the frequency and percentage of categorical factors such as gender, skin type, and face mask to compare the results. An outcome categorical variable pattern was shown using tables and bar graphs. We employed independent t-tests and ANOVA to compare numerical and categorical variables for inferential statistical purposes, and chi-square tests were used to analyze categorical variables. A P-value of less than 0.05 was considered significant.

Ethical Consideration: An ethical clearance has been provided by the institutional review board (IRB) at King Abdullah International Medical Research Center, National Guard Health Affairs, Jeddah, Saudi Arabia (IRB approval number: NRJ21J/029/02), prior to conducting the study. Thus, the anonymity and privacy of the responders were ensured. 

## Results

Analyzed results included 446 individuals, of which 292 (65.5%) were females, all the participants were from the Western region. The demographic variables considered are given in Table 1.

**Table 1:** The sociodemographic characteristics of the participants. n = Number of participants; IQR = Interquartile range

	n	Median	IQR
Age in years:	446	30	23-43
Weight in kg:	446	69	58-85
Height in cm:	446	164	158-170
BMI in kg/m^2^:	445	25.5	21.8-29.7
		n=446	%
Gender:			
	Male	154	34.5
	Female	292	65.5
Region:			
	Western	446	100

Table 2 demonstrates the skin types among participants; more than one-third (36.8%, n=164) had mixed skin type and 20.9% (n=93) of the population were diagnosed with a skin condition in the past two years.

**Table 2:** Skin type among the participants. n = Number of participants

	n	%
In males, do you have a beard or mustache? (n=154)		
Mustache	28	18.2
Beard	2	1.3
Mustache & Beard	115	74.7
None	9	5.8
What is your skin type? (n=446)		
Mixed	164	36.8
Greasy	82	18.4
Normal	133	29.8
Dry	24	5.4
Sensitive	43	9.6

**Figure 1:**
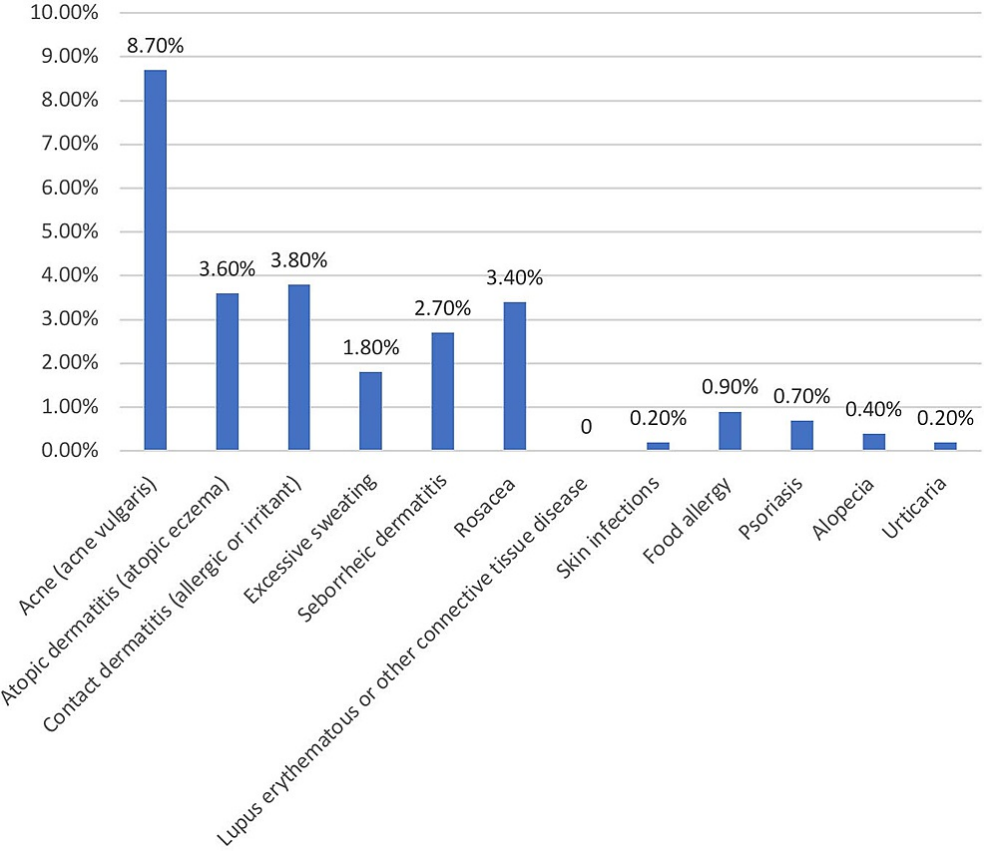
Skin conditions among the participants (n=446) n = Number of participants

As displayed in (Figure 1), acne vulgaris was the highest documented pre-existing skin condition. Around half of the patients (52.7%, n=49) who were diagnosed with pre-existing skin condition reported that their condition did not improve after wearing masks, in fact, only 10.8% (n=10) stated that their condition has improved. On the contrary, 46.2% (n:43) had experienced an exacerbation of their condition. Both duration and mask-related results are summarized in (Table 3). 

**Table 3:** The effect of wearing a mask on pre-existing skin conditions and the habits of wearing mask among the participants. n = Number of participants

		n	%
Is your condition improved with or after wearing a mask? (n=93)			
	Yes	10	10.8
	No	49	52.7
	I did not notice	34	36.6
Have you noticed the exacerbation of the skin condition with or after wearing a mask? (n=93)			
	Yes	43	46.2
	No	24	25.8
	I did not notice	26	28.0
After how long of wearing the face mask you had experienced a flare-up (n=46)			
	<1 hour	10	21.7
	1-6 hours	15	32.6
	6-24 hours	1	2.2
	1-7 days	8	17.4
	1 week-1 month	7	15.2
	> 1 month	5	10.9
After you have experienced flare-up, have you changed your mask type? (n=46)			
	Yes	27	58.7
	No	19	41.3
What type of face mask do you wear? (n=445)			
	Surgical mask	368	82.7
	Respirator mask with filtering face piece	8	1.8
	Cloth mask	33	7.4
	KN95	5	1.1
	Face shield with mask	3	0.7
	Niqab with surgical mask	23	5.2
	Niqab with cloth mask	5	1.1
What is the material of your cloth mask? (n=38)			
	100% cotton	4	10.5
	Partially cotton	7	18.4
	Polyester	6	15.8
	I do not know	21	55.3
How many times you wash your cloth mask? (n=38)			
	After every use	4	10.5
	Every 2 to 3 days	16	42.1
	Every week	9	23.7
	Not at all	9	23.7
Do you use a double mask, for example, cloth mask with surgical mask? (n=446)			
	Yes	0	0
	No	446	100.0
For how long do you wear a face mask per day? (n=446)			
	Up to 1 hour	51	11.4
	1-2 hours	130	29.1
	2-5 hours	146	32.7
	>5 hours	117	26.2
	I do not wear a mask	2	0.4
Where do you spend your time mostly wearing a mask? (n=446)			
	Indoor	360	80.7
	Outdoor	86	19.3

**Figure 2:**
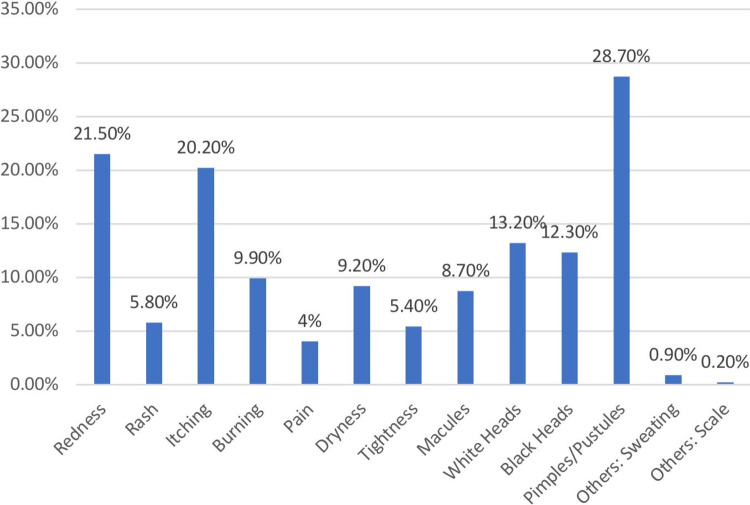
Skin changes among the participants after wearing face mask (n=446) n = Number of participants

The participants were asked about the types of products they use for their skin. Moisturizing products were reported by 37% (n=165), cosmetics 29.6% (n=132), sunblock 23.3% (n=104), medical cream 0.2% (n=1) and 48.4% (n=216) do not use any medications or products for their skin (Table 4). Out of the total, 41.7% (n=186) of the participants reported acquiring new skin changes during the pandemic after using the mask. Participants were asked about the specific skin conditions which included redness, rash itching, burning, pain, dryness, tightness, and others. The results are summarized in Figure 2.

**Table 4:** The use of skin-care products among the participants. n = Number of participants

		n=446	%
Cosmetics			
	Yes	132	29.6
	No	314	70.4
Moisturizing products			
	Yes	165	37.0
	No	281	63.0
Sunblock			
	Yes	104	23.3
	No	342	76.7
None			
	Yes	216	48.4
	No	230	51.6
Others: Medical creams			
	Yes	1	0.2
	No	445	99.8
Others: Minoxidil			
	Yes	0	0.0
	No	446	100
Do you experience excessive sweating problems under the face mask?			
	Yes	184	41.3
	No	262	58.7

The duration of symptoms and the duration of mask usage before the appearance of skin symptoms varied among the participants. The results of the duration and sites of the symptoms are shown in Table 5. Self-management of the participants in response to their skin conditions ranged from the use of cold water or ice to topical medical treatment like antibiotic cream or corticosteroids. See the related results in Table 6. 

**Table 5:** The duration and sites of the skin-related symptoms among the participants. n = Number of participants

		n	%
When did you notice the appearance of skin symptoms after wearing a mask? (n=186)			
	<1 hour	22	11.8
	1-6 hours	36	19.4
	6-24 hours	29	15.6
	1-7 days	27	14.5
	1 week-1 month	51	27.4
	> 1 month	21	11.3
How long did the skin changes continue after wearing a mask? (n=186)			
	<24 hours	39	21.0
	1 -7 days	78	41.9
	1-2 weeks	26	14.0
	> 2 weeks	43	23.1
Cheeks (n=446)			
	Yes	140	31.4
	No	306	68.6
Around the lips (n=446)			
	Yes	81	18.2
	No	365	81.8
Bridge of the nose (n=446)			
	Yes	72	16.1
	No	374	83.9
Neck (n=446)			
	Yes	18	4.0
	No	428	96.0
Chin (n=446)			
	Yes	94	21.1
	No	352	78.9
Jaw line (n=446)			
	Yes	44	9.9
	No	402	90.1
Eyes (n=446)			
	Yes	7	1.6
	No	439	98.4
Forehead (n=446)			
	Yes	7	1.6
	No	439	98.4
Have you ever stopped wearing the face mask after you have noticed skin problems caused by them? (n=186)			
	Yes	6	3.2
	No	131	70.4
	Sometimes	49	26.3

**Table 6:** Participants' management for the mask-related facial dermatosis. n = Number of participants

		n=446	%
Moisturizer			
	Yes	74	16.6
	No	372	83.4
Topical medical treatment (for example: Fucidine, topical corticosteroids, topical antibiotics, Differin, Acretin)			
	Yes	78	17.5
	No	368	82.5
Cosmetics (vitamin C, hyaluronic acid, toner, Face masks)			
	Yes	48	10.8
	No	398	89.2
Have not used something			
	Yes	37	8.3
	No	409	91.7
Cold water or ice			
	Yes	44	9.9
	No	402	90.1
Non-medical (eg: Honey, Aloe Vera Gel, myrrh, corn starch, rose water)			
	Yes	15	3.4
	No	431	96.6

The outcome variable was the skin changes after the use of face masks during the pandemic of COVID-19 which was measured using the question “Since the beginning of COVID-19 pandemic, have you noticed any skin changes associated with face mask usage?”. 'Yes' or 'No' answer was measured for the association as a binary outcome using Chi-square and Fisher exact tests. The results showed statistically significant associations with skin type (P<0.001), improvement and exacerbation of a pre-existing condition (P<0.001), duration of wearing the face mask (P<0.001). See the results obtained in Table 7. Nevertheless, the same outcome variable was tested for significance with symptoms indicated by the participants. The results showed various significant associations which are shown in Table 8.

**Table 7:** The association between skin changes after the use of face mask during the pandemic and related risk factors. *Chi-square test; **Fisher exact test; n = Number of participants

		Yes		No		p
		n	%	n	%	
Do you have a beard or mustache?						0.813**
	Mustache	6	23.1.%	22	17.2%	
	Beard	0	0.0%	2	1.5%	
	Mustache & Beard	19	73.1%	96	75%	
	None	1	3.8%	8	6.3%	
What is your skin type?						<0.001*
	Mixed	76	46.3%	88	53.7%	
	Greasy	41	50.0%	41	50.0%	
	Normal	37	27.8%	96	72.2%	
	Dry	5	20.8%	19	79.2%	
	Sensitive	27	62.8%	16	37.2%	
Is your condition improved with or after wearing a mask?						<0.001*
	Yes	4	40.0%	6	60.0%	
	No	42	85.7%	7	14.3%	
	I did not notice	6	17.6%	28	82.4%	
Have you noticed the exacerbation of the skin condition with or after wearing a mask?						<0.001*
	Yes	41	95.3%	2	4.7%	
	No	6	25.0%	18	75.0%	
	I did not notice	5	19.2%	21	80.8%	
After how long of wearing the face mask you had experience flare up						0.151**
	<1 hour	7	70.0%	3	30.0%	
	1-6 hours	15	100.0%	0	0.0%	
	6-24 hours	1	100.0%	0	0.0%	
	1-7 days	7	87.5%	1	12.5%	
	1 week-1 month	7	100.0%	0	0.0%	
	> 1 month	4	80.0%	1	20.0%	
For how long do you wear a face mask per day?						<0.001*
	Up to 1 hour	9	17.6%	42	82.4%	
	1-2 hours	40	30.8%	90	69.2%	
	2-5 hours	67	45.9%	79	54.1%	
	>5 hours	70	59.8%	47	40.2%	
	I do not wear a mask	0	0.0%	2	100.0%	

**Table 8:** Association between skin changes after mask use during the pandemic and related symptoms. *Chi-square test; **Fisher exact test; n = Number of participants

		Yes		No		P
		n	%	n	%	
Redness						<0.001*
	Yes	96	100.0%	0	0.0%	
	No	90	25.7%	260	74.3%	
Rash						<0.001*
	Yes	25	96.2%	1	3.8%	
	No	161	38.3%	259	61.7%	
Itching						<0.001*
	Yes	90	100.0%	0	0.0%	
	No	96	27.0%	260	73.0%	
Burning						<0.001*
	Yes	44	100.0%	0	0.0%	
	No	142	35.3%	260	64.7%	
Pain						<0.001*
	Yes	18	100.0%	0	0.0%	
	No	168	39.3%	260	60.7%	
Dryness						<0.001*
	Yes	41	100.0%	0	0.0%	
	No	145	35.8%	260	64.2%	
Tightness						<0.001*
	Yes	24	100.0%	0	0.0%	
	No	162	38.4%	260	61.6%	
Macules						<0.001*
	Yes	39	100.0%	0	0.0%	
	No	147	36.1%	260	63.9%	
White Heads						<0.001*
	Yes	59	100.0%	0	0.0%	
	No	127	32.8%	260	67.2%	
Black Heads						<0.001*
	Yes	55	100.0%	0	0.0%	
	No	131	33.5%	260	66.5%	
Pimples / pustules						<0.001*
	Yes	128	100.0%	0	0.0%	
	No	58	18.2%	260	81.8%	
Sweating						0.030**
	Yes	4	100.0%	0	0.0%	
	No	182	41.2%	260	58.8%	
Loss of facial hair						Cannot be calculated
	Yes	0	0.0%	0	0.0%	
	No	186	41.7%	260	58.3%	
Scale						0.417**
	Yes	1	100.0%	0	0.0%	
	No	185	41.6%	260	58.4%	

## Discussion

The goal of this study was to discover whether there was a link between skin dermatosis and the use of facial masks. Among our sample, only 20.9% (n=93) of participants have been diagnosed with skin conditions before the beginning of the pandemic. Acne was the most reported pre-existing skin condition among our participants by 8.7% (n=39). The other states are reported though not in high percentages. As reported by the participants, wearing a facial mask did not play many roles in improving their skin conditions. On the other hand, wearing a mask for an extended period caused flare-ups that may result in changing the type of the masks as 58.7% (n=27) of those who have experienced flare-ups have changed their mask type. People who use cloth masks also washed their masks after a duration of use, leading to facial dermatosis in the long run. Wearing a mask for 2-5 hours a day indoors proved to be one of the contributory factors of facial dermatosis [13,14].

The current study investigated the facial dermatosis problem among the general population rather than the majority of previous studies which focused on health care providers alone. We noticed some differences in the findings between the general population and health care workers in two aspects. First, pimples and pustules were the most common skin changes associated with face-mask use in the general population [14]. In comparison to our findings, redness and itchiness were the most popular skin changes after wearing a face mask among health care workers [13,15]. Second, the cheek, chin, and around lips were the most severely impacted areas unlike nasal bridge in health care workers, suggesting that prevention efforts should be directed at these regions [10,16]. These variations may be due to the fact that healthcare workers wear masks for a longer duration than others [13].

Most of the participants took more than a week to notice the skin changes, about 27.4% out of 186 people. These changes were noticed after wearing masks, which can be explained as that the continuous use of face masks caused the skin changes. Most of the participants did not eliminate using the masks even after these changes occurred but used products such as the topical medical treatment and cosmetics to alleviate their symptoms. Although most people in the research never noticed these changes after wearing masks, it is clear that there is a close association between face masks and facial dermatosis [15]. The skin conditions mainly occurred after wearing a mask for more than a week than 5 hours a day and were most likely caused by wearing a surgical mask since 82.7% (n=368) of the people questioned were using surgical masks. Wearing a surgical mask showed a higher risk of adverse skin reactions compared to a cloth mask [13,14]. People who were most likely to have skin dermatosis due to face mask use were people with sensitive skin type by 62.8% (n=27) followed by greasy skin 50% (n=41) and mixed skin 46.3% (n=76). The etiology of dermatological diseases linked with masks involves friction, humidity, and mechanical pressure. Long-term usage of the masks was reported to cause mechanical skin damage, maceration, abrasion, erythema, desquamation, itching, and acne [16].

In line with past studies on skin dermatoses, this study found that wearing masks for an extended period exacerbated conditions such as acne on the face [12,16]. Another typical symptom to consider is itching, particularly noticeable on the cheeks and upper arms [17]. An occlusive effect hampered the skin's hydration and irritated pilosebaceous gland ducts owing to the increased warmth and moisture of the facial skin induced by expired air and sweating [13]. 

Limitations

There are a few limitations in our study, one of the drawbacks of this study is that it has a relatively small sample size to represent one region of the kingdom so our findings and observations are limited in their generalizability to the whole population. The majority of the participants were females. Data collection was an online self-administered questionnaire used in this cross-sectional study which is very sensitive to recall bias.

## Conclusions

In conclusion, skin disorders such as new and unpleasant dermatoses and the worsening of pre-existing skin conditions result from the prolonged mask use during the COVID-19 pandemic. In the current study, approximately half of the participants indicated that their pre-existing skin conditions were aggravated by wearing masks. Out of the total, 41.7% of participants indicated that they have noticed skin changes after wearing face masks during the pandemic. Out of all mask types, the surgical mask was the most common contributor to facial dermatosis. Additionally, the duration of wearing masks and facial dermatosis had a significant correlation.
